# The Gut–Heart–Kidney Axis in Heart Failure: Trimethylamine N-Oxide and Beyond—A State-of-the-Art Review

**DOI:** 10.3390/biomedicines14092054

**Published:** 2026-09-12

**Authors:** Ismaila Ajayi Yusuf, Solomon Anighoro, Abdullah Sultany, Sheeza Nawaz, Ayush Adhikari, Shubhendu Bajpai, Ashraf Ullah, Arundhati Sharma, Sahil Grover, Naga Sumanth Reddy Gopireddy, Amlish Gondal, Michelle Bernshteyn, Subash Ghimire

**Affiliations:** 1 Department of Internal Medicine, Guthrie Robert Packer Hospital, Sayre, PA 18840, USAsheeza.nawaz@guthrie.org (S.N.); sahil.grover@guthrie.org (S.G.);; 2 Department of Gastroenterology, Guthrie Robert Packer Hospital, Sayre, PA 18840, USA

**Keywords:** trimethylamine N-oxide, heart failure, gut microbiota, gut–heart–kidney axis, prognostic biomarker, HFrEF, HFpEF, trimethyllysine, phenylacetylglutamine, metabolite panel

## Abstract

Trimethylamine N-oxide (TMAO), a gut microbiota-derived metabolite of dietary choline and L-carnitine, has emerged as a leading molecular mediator of the gut–heart–kidney axis in heart failure (HF). This state-of-the-art narrative review synthesizes evidence from 14 observational studies (13 prospective cohorts and one cross-sectional analysis), encompassing a heterogeneous range of HF settings, including established chronic HF, acute decompensated HF, incident HF in community cohorts, and subclinical myocardial injury. Across these populations, elevated circulating TMAO has been associated with adverse outcomes, including mortality, rehospitalization, and major adverse cardiovascular events, although the strength and consistency of associations vary by HF phenotype, renal function status, and population ancestry. The prognostic independence of TMAO is attenuated after adjusting for renal function in several cohorts, reflecting both obligatory renal clearance and a potentially bidirectional relationship with kidney injury. TMAO was not reduced during neurohormonal GDMT uptitration in the BIOSTAT-CHF study, suggesting that gut microbiota dysbiosis may represent a pathophysiological axis not reached by current HF treatment. The field has evolved through three thematic stages: TMAO as a single prognostic biomarker, TMAO within cardiorenal pathophysiology, and multimetabolite and multipathway risk profiling. Beyond the TMAO pathway, emerging evidence for phenylacetylglutamine (PAGln), short-chain fatty acids (SCFAs), and protein-bound uremic toxins as parallel gut-derived cardiovascular mediators supports a multidimensional metabolite profiling approach. Several cohorts suggest that selected multimetabolite panels may provide additional prognostic information beyond TMAO alone, although their composition, calibration, and external validity remain uncertain. Population-specific variation in TMAO levels and prognostic thresholds complicates universal clinical application. Without human interventional data demonstrating that lowering TMAO improves cardiovascular outcomes, TMAO remains a prognostic risk marker rather than a validated clinical target. Clinical translation requires resolving the renal confounding problem, validating multimetabolite panels across diverse populations, and conducting intervention trials targeting the gut–heart–kidney axis.

## 1. Introduction

Heart failure affects more than 64 million people worldwide and remains a leading cause of cardiovascular mortality, with five-year survival rates comparable to many malignancies [1]. Despite advances in GDMT targeting neurohormonal activation through renin–angiotensin–aldosterone system (RAAS) inhibition, beta blockade, and mineralocorticoid receptor antagonism, risk stratification beyond natriuretic peptides and cardiac troponins remains an active clinical priority [2]. These conventional biomarkers reflect hemodynamic stress and myocardial injury, but capture little of the pathophysiology arising from systemic metabolic disturbance, leaving a substantial proportion of high-risk patients inadequately characterized.

The gut microbiota has emerged as a cardiovascular endocrine organ of substantial relevance, and its relationship with the failing heart has evolved through three thematic stages that structure this review. These stages overlap in time and are defined by conceptual advance rather than strict chronological boundaries.

Stage 1—TMAO as a Single Prognostic Biomarker. Wang et al. established in 2011 that gut microbial metabolism of dietary phosphatidylcholine generates trimethylamine N-oxide (TMAO), a molecule that promotes atherosclerosis and predicts cardiovascular events in humans [3]. Tang et al. extended this observation to chronic HF in 2014, demonstrating that elevated TMAO independently predicted five-year mortality in stable HF patients, establishing the gut–heart connection in an established HF population for the first time [4].

Stage 2—TMAO within Cardiorenal Pathophysiology. Subsequent cohort studies revealed that TMAO prognostic associations were frequently attenuated after renal function adjustment, prompting investigation of whether TMAO actively promotes kidney injury rather than merely accumulating due to impaired clearance [5]. Experimental evidence confirmed that TMAO induces renal tubulointerstitial fibrosis, implicating a gut–heart–kidney triad rather than a simple gut–heart axis [6]. The pivotal BIOSTAT-CHF study demonstrated in 2019 that TMAO levels were not reduced during GDMT uptitration, suggesting the gut microbiota axis may operate independently of neurohormonal mechanisms [7].

Stage 3—Multimetabolite and Multipathway Risk Profiling. The recognition that TMAO alone incompletely captures gut dysbiosis risk motivated investigation of the broader choline–carnitine–TMAO pathway. Studies demonstrated that upstream precursors, including trimethyllysine (TML), acetyl-L-carnitine (AcCarn), crotonobetaine, and gamma-butyrobetaine (GBB), may contribute independent prognostic information and that multimetabolite panels may provide greater prognostic information than single-marker TMAO in selected cohorts [8,9,10]. Concurrently, metabolites from entirely distinct gut microbial pathways, primarily PAGln, SCFAs, and protein-bound uremic toxins, have emerged as parallel cardiovascular mediators that together justify a multidimensional gut metabolite profiling approach, though the evidence base for these metabolites remains less mature than for TMAO.

This state-of-the-art review synthesizes evidence from 14 observational studies, summarized in Table 1, appraises the renal confounding problem, examines the HFrEF-versus-HFpEF controversy, and proposes a framework for clinical translation. Four analytical questions are kept distinct throughout: whether TMAO is associated with clinical outcomes; whether TMAO measurement improves risk prediction; whether TMAO causally contributes to HF progression; and whether reducing TMAO would improve outcomes. These questions carry different evidence standards and have different answers in the current literature.

## 2. Literature Search and Review Methodology

This review was conducted as a state-of-the-art narrative review rather than a systematic review or meta-analysis and was therefore not registered in PROSPERO. The Scholarly Article Reporting Standards for Narrative Reviews (SANRA) framework was used as a guiding checklist for methodological transparency.

A structured literature search was performed in PubMed, Embase, and Web of Science from January 2011 through April 2025. The following MeSH terms were applied: trimethylamine N-oxide, gastrointestinal microbiome, heart failure, cardiorenal syndrome, kidney diseases, metabolomics, and cardiovascular diseases. Free-text keywords included TMAO, gut microbiota, gut microbiome, heart failure, cardiorenal syndrome, phenylacetylglutamine, indoxyl sulfate, p-cresyl sulfate, short-chain fatty acids, HFrEF, HFpEF, and cardiovascular outcomes. MeSH and free-text terms were combined using AND and OR Boolean operators across title, abstract, and keyword fields. No language restrictions were applied.

Explicit inclusion criteria for the primary evidence base were: (1) prospective cohort or observational design; (2) measurement of circulating TMAO or related gut-derived metabolites; (3) reporting of HF-related outcomes including mortality, hospitalization, or incident HF; and (4) human participants. Cross-sectional studies were excluded from the primary evidence base, but were included when they provided mechanistically relevant evidence not available from prospective designs. Mechanistic studies, meta-analyses, and translational investigations were included where directly relevant to biological pathways, renal interactions, or therapeutic implications, with selection based on relevance to the three thematic stages described herein. Studies addressing PAGln, SCFAs, and uremic toxins were identified through the same search strategy using the free-text keywords listed above and were selected based on direct relevance to cardiovascular outcomes in HF or cardiorenal populations.

Reference lists of key articles were manually screened to identify additional relevant publications. Where the same cohort contributed multiple publications (for example, the Leicester AHF cohort contributing Suzuki 2016 [13] and Israr 2021 [8], and the CHS/MESA cohort contributing Tang 2024 [19]), each publication was treated as a separate entry in Table 1 when it reported distinct metabolites, analytical approaches, or outcome data, but they were not treated as independent participant contributions when estimating total unique participants. Fourteen prospective observational studies and one cross-sectional analysis constituted the principal evidence base. Findings were interpreted in the context of sample size, follow-up duration, HF phenotype, analytical platform, extent of renal adjustment, and consistency across independent cohorts.

**Figure 1:**
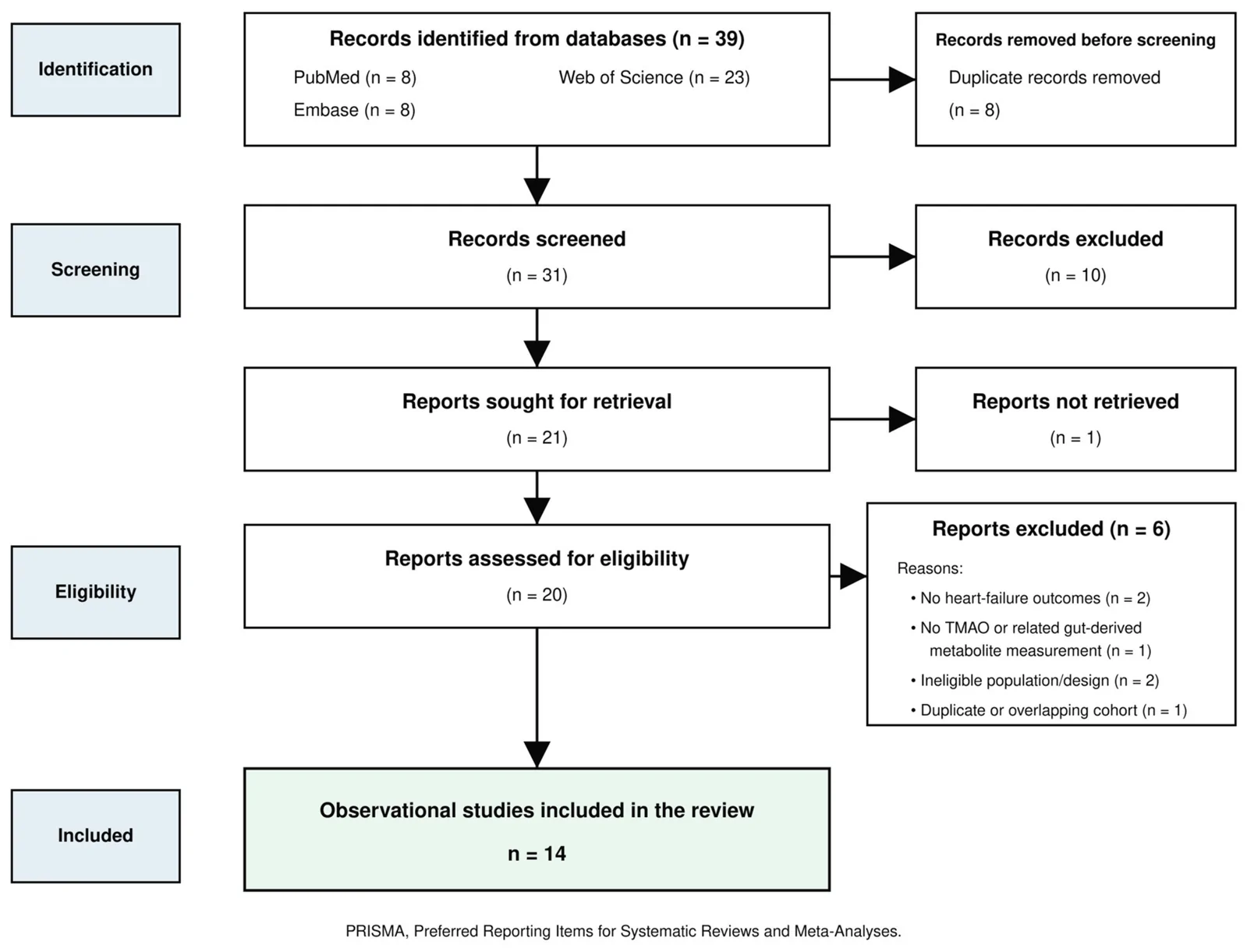
PRISMA flowchart.

No formal risk-of-bias assessment tool, such as GRADE or the Newcastle–Ottawa Scale was applied, consistent with the narrative nature of the review. However, key methodological limitations, including single-center design, heterogeneity of HF definitions, variation in ejection fraction thresholds for HFpEF, variation in follow-up duration, and the observational nature of all included studies, are discussed throughout the manuscript where relevant. Figure 1 provides a flowchart of the study identification and selection process.

## 3. TMAO Biochemistry and the Gut–Heart–Kidney Axis

### 3.1. Dietary Precursors and Microbial Generation

TMAO is the terminal oxidation product of a multistep metabolic pathway initiated by dietary intake. Primary precursors, including phosphatidylcholine, choline, L-carnitine, betaine, and TML, are abundant in red meat, egg yolks, fish, and dairy products. In the intestinal lumen, gut microbial taxa, including members of Firmicutes, Proteobacteria, and Actinobacteria, possess trimethylamine lyase enzymes encoded by the cut and gbu gene clusters, which cleave trimethylamine (TMA) from precursor molecules [3]. (Gene cluster names: cut and gbu are used here as established nomenclature for the choline utilization and gamma-butyrobetaine gene clusters, respectively.) L-carnitine undergoes sequential conversion through GBB and crotonobetaine before generating TMA, while choline is converted more directly via the cut pathway, representing a quantitatively important substrate particularly in populations with high animal product consumption [20].

### 3.2. Hepatic Oxidation, Renal Excretion, and the Gut–Heart–Kidney Triad

**Figure 2:**
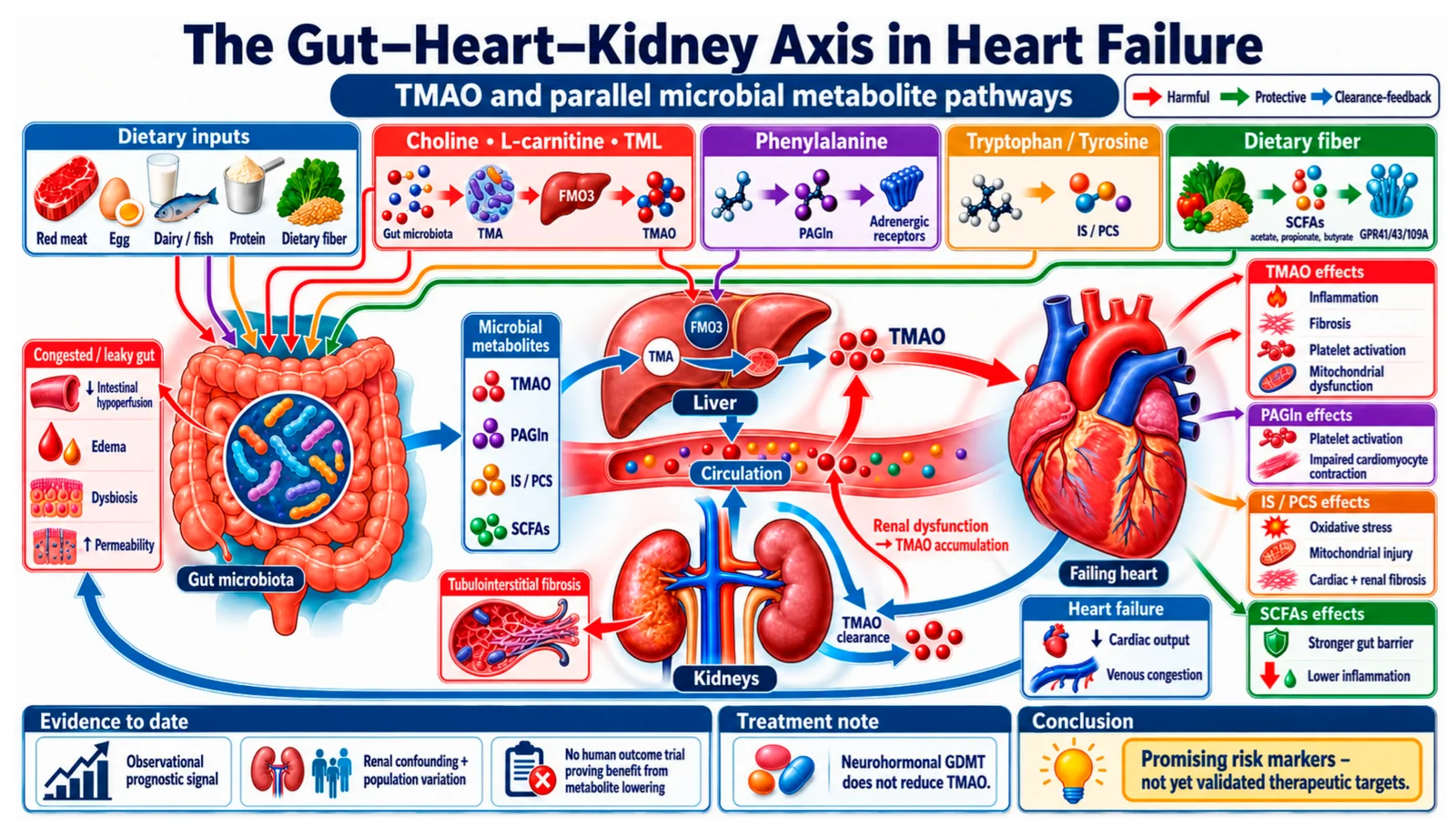
Central illustration of the gut–heart–kidney axis in heart failure.

Absorbed TMA is transported to the liver via the portal circulation, where it undergoes oxidation to TMAO by hepatic flavin-containing monooxygenase 3 (FMO3), the rate-limiting enzymatic step in TMAO biosynthesis [3]. FMO3 activity is subject to genetic polymorphism, hormonal regulation, and dietary modulation, contributing to substantial inter-individual variation in circulating TMAO even under controlled dietary conditions [21]. TMAO is subsequently excreted renally, with urinary excretion representing the primary clearance route. This renal dependence is central to the gut–heart–kidney triad: TMAO promotes renal tubulointerstitial fibrosis in preclinical models, impairing clearance and potentially further elevating circulating levels, which in turn may promote additional cardiac and renal injury [6]. In HF, reduced cardiac output and elevated venous pressures produce splanchnic congestion, mucosal edema, and intestinal hypoperfusion, driving gut microbiota dysbiosis, increasing gut permeability, and potentially elevating TMAO production, which may feed back to worsen cardiac and renal function (Figure 2) [22].

## 4. Mechanisms Linking TMAO to Cardiac and Renal Injury

### 4.1. Cardiac and Vascular Mechanisms

TMAO exerts potentially proatherogenic effects through upregulation of macrophage scavenger receptors CD36 and SR-A, impaired reverse cholesterol transport, and promotion of foam cell formation in preclinical models [3]. It directly modulates platelet calcium signaling, potentially enhancing thrombosis risk, a mechanism with particular relevance to ischemic HF following myocardial infarction. At the myocardial level, preclinical and review-level evidence supports TMAO as a potential contributor to HF progression through multiple pathways: TGF-beta-mediated cardiac hypertrophy and interstitial fibrosis, supported by preclinical models [9]; endothelial inflammation through reactive oxygen species (ROS)–TXNIP (thioredoxin-interacting protein)–NLRP3 inflammasome activation via SIRT3 (sirtuin 3)–SOD2 (superoxide dismutase 2)–mitochondrial ROS signaling demonstrated in vascular endothelial cells [23]; and impaired mitochondrial fatty acid oxidation in cardiomyocytes (cell-based evidence) [23]. These mechanistic claims derive from cell-based and animal-model studies. Human mechanistic evidence in HF populations remains limited. Organ et al. demonstrated in a murine transverse aortic constriction model that both dietary TMAO withdrawal and pharmacological inhibition of gut microbial choline TMA lyase with iodomethylcholine improved left ventricular function and attenuated adverse ventricular remodeling compared with TMAO-fed controls [24]. This establishes preclinical biological plausibility for the clinical associations in HF cohorts, though human interventional confirmation that lowering TMAO improves cardiac outcomes is currently lacking.

### 4.2. Renal Mechanisms and Upstream Precursor Toxicity

TMAO promotes renal tubulointerstitial fibrosis and progressive glomerular filtration rate decline in animal models, generating the cardiorenal cycle central to this review [6]. Upstream precursors contribute potential additional toxicity through distinct mechanisms. The TML derivative trimethyl-5-aminovaleric acid (TMAVA) inhibits fatty acid oxidation in cardiac myocytes via suppression of BBOX (gamma-butyrobetaine hydroxylase)-dependent carnitine synthesis, accelerating cardiac hypertrophy and dysfunction in murine models, with effects reversed by exogenous carnitine supplementation (animal-model evidence) [25]. Crotonobetaine was independently associated with incident HF in large prospective cohorts even after renal function adjustment [19]; however, describing this as implying a mechanism entirely distinct from TMAO accumulation overstates what observational independence from a statistical covariate can establish about underlying biology. The mechanistic basis for crotonobetaine’s independent association remains incompletely characterized.

## 5. Clinical Evidence Across Three Thematic Stages

### 5.1. Stage 1—TMAO as a Single Prognostic Biomarker

Tang et al. established the prognostic relevance of TMAO in 720 stable CHF patients from the Cleveland Clinic, demonstrating a 3.4-fold increase in five-year all-cause mortality in the highest-versus-lowest TMAO quartile (HR 3.42, 95% CI 2.24–5.23), with the combination of elevated TMAO and BNP conferring 5.7-fold mortality risk [4]. Trøseid et al. extended these observations to 155 stable HF patients in Oslo, making a critical etiology-specific observation: TMAO followed a gradient from highest in ischemic HF through stable coronary artery disease to lowest in non-ischemic dilated cardiomyopathy, persisting after adjustment for renal function [5]. Only TMAO, not choline or betaine, predicted transplant-free survival in this cohort, suggesting that the final oxidative step captures prognostic information not reflected by precursor accumulation alone.

Schuett et al. reported the first phenotype-stratified TMAO analysis from the LURIC cohort of 823 HF patients, including 395 HFpEF patients, making it the largest HFpEF-specific dataset in the TMAO literature [12]. TMAO was associated with all-cause mortality in HFrEF and cardiovascular mortality, with associations surviving full multivariable adjustment, while TMAO was not significantly associated with mortality in HFpEF. In HFrEF, TMAO added 37.7% improvement in net reclassification (NRI) beyond NT-proBNP alone; however, NRI values can appear favorable even when the actual improvement in clinical decision-making is modest, and changes in the C-statistic or time-dependent AUC were not reported. Senthong et al. provided complementary mechanistic evidence in 134 stable high-cardiovascular-risk participants from the CORE-Thailand registry. This cross-sectional analysis of a non-HF population demonstrated that TMAO correlated with high-sensitivity cardiac troponin T (hs-cTnT; r = 0.54) and independently predicted subclinical myocardial damage after eGFR adjustment (OR 1.58) [11], providing a mechanistic bridge between the atherosclerosis and HF literatures.

### 5.2. Stage 2—TMAO Within Cardiorenal Pathophysiology

BIOSTAT-CHF (Suzuki et al.) enrolled 2234 patients with new or worsening HF across 69 European centers [7]. TMAO was independently associated with mortality after adjustment, including eGFR, with NRI of 16.8%. Over 9 months of GDMT uptitration, BNP fell substantially and NYHA class improved markedly, but TMAO did not decrease: instead, it slightly increased regardless of ACE inhibitor, ARB, or beta-blocker dose escalation. This finding suggests the gut microbiota axis may be driven by mechanisms not modulated by neurohormonal pathways, though it does not establish mechanistic independence and does not exclude shared downstream effectors. Patients with persistently high TMAO at both enrollment and follow-up carried more than double the mortality risk, while a single low measurement at either time point conferred low-risk status.

The Leicester AHF cohort (Suzuki et al.) enrolled 972 patients with acute HF, demonstrating that TMAO was associated with one-year all-cause mortality and the death-or-rehospitalization composite [13]. Patients with both elevated TMAO and NT-proBNP had more than three times the risk of death or rehospitalization compared with patients with neither elevated. TMAO added predictive value when incorporated into ADHERE, OPTIMIZE-HF, and GWTG-HF clinical risk scores; however, the association with mortality was lost after adjustment for eGFR due to severe multicollinearity (VIF 16.2), illustrating the analytical challenge of disentangling TMAO effects from renal function in this population. Zhou et al. demonstrated in 1208 post-MI CHF patients that TMAO remained independently associated with MACEs and all-cause mortality after full adjustment, including eGFR and hsCRP [16]. Integrated discrimination improvement (IDI) was +12.6% and NRI was +9.5%, with the same caveats regarding NRI interpretation applying here.

The HFpEF controversy was addressed by two studies, both with significant sample size limitations. Kinugasa et al. demonstrated in 146 Japanese HFpEF patients from a single center that elevated TMAO at discharge independently predicted cardiac death and HF hospitalization, with the prognostic effect amplified substantially in malnourished patients [17]. The DIAMONDHFpEF study (Salzano et al.) enrolled 118 HFpEF patients from a single UK center and demonstrated that TMAO above 5 µmol/L conferred fourfold increased composite endpoint risk at 18 months, surviving eGFR adjustment [14]. While these two hypothesis-generating studies suggest possible prognostic value of TMAO in HFpEF, the null result from the LURIC cohort (n = 395), the largest HFpEF-specific dataset available, carries greater statistical weight: caution is required before concluding that TMAO is a validated prognostic marker in HFpEF. A prospective multicenter HFpEF cohort study with prespecified panel analysis is needed.

### 5.3. Stage 3—Multimetabolite and Multipathway Risk Profiling

Israr et al. extended the Leicester AHF analysis to a ten-metabolite panel in 806 patients, revealing a temporal dissociation of clinical importance: acetyl-L-carnitine (AcCarn) dominated short-term prognostication, achieving the highest AUC for in-hospital mortality and substantially outperforming TMAO at that time point, while TMAO emerged as the dominant predictor of one-year outcomes [8]. Carnitine-pathway metabolites improved NRI for in-hospital mortality when added to established scores by up to 56.9%, whereas TMAO did not. These NRI values require cautious interpretation, as described above. This temporal dissociation—carnitine metabolites for acute triage and TMAO for post-discharge risk stratification—has direct potential implications for clinical biomarker strategy, though external validation is needed before incorporation into practice.

Zong et al. demonstrated that TML was independently associated with cardiovascular death and all-cause mortality in 471 stable CHF patients after full adjustment, including TMAO itself [18]. Tang et al. analyzed six metabolites in 11,768 CHS and MESA participants over a median follow-up of 15.9 years, during which 2102 incident HF cases occurred. TMAO, choline, and crotonobetaine were each independently associated with incident HF, with choline retaining association after eGFR adjustment. Associations appeared generally stronger among Black and Hispanic/Latino versus White adults, though interaction tests were not statistically significant for any metabolite [19]. Yazaki et al. conducted a tri-ethnic analysis in 1087 AHF patients, demonstrating that elevated TMAO predicted death or rehospitalization in Caucasian patients, but not in South Asian or Japanese patients at equivalent thresholds [15]. Differences in renal function, assay conditions, medication use, and dietary patterns across populations may contribute to this heterogeneity beyond ethnicity alone (Table 2). Suzuki et al. validated the panel approach in 229 Chinese AHF patients, demonstrating that a five-marker panel was associated with higher hazard ratios for mortality than single-marker TMAO in this cohort; however, C-statistic improvement and external validation were not reported, and hazard ratios from differently constructed panels should not be compared directly as measures of predictive performance [10].

### 5.4. Beyond the TMAO Pathway: PAGln, Short-Chain Fatty Acids, and Uremic Toxins

The title of this review deliberately extends beyond TMAO to encompass gut-derived metabolites from distinct microbial pathways that may broaden the mechanistic and prognostic scope of the gut–heart–kidney axis in HF.

PAGln is a phenylalanine-derived gut microbial metabolite that has emerged as a TMAO-independent cardiovascular risk factor with potential relevance to HF. Nemet et al. identified PAGln through untargeted metabolomics in over 4000 subjects and demonstrated that it promotes platelet activation and thrombosis through alpha-2A, alpha-2B, and beta-2 adrenergic receptor signaling (human observational and preclinical mechanistic evidence) [26]. Romano et al. subsequently demonstrated in two independent clinical cohorts that PAGln was dose-dependently associated with HF presence and severity independently of traditional risk factors and renal function, and that PAGln directly fostered HF-relevant phenotypes, including decreased cardiomyocyte sarcomere contraction and increased BNP gene expression (cell-based and human observational evidence) [27]. Tang et al. demonstrated in two large stable HF cohorts that the highest PAGln quartile was associated with a 3.09-fold increased five-year mortality risk, with prognostic value independent of TMAO levels [28]. Saha et al. identified PAGln as an endogenous negative allosteric modulator of beta-2 adrenergic receptors (beta-2ARs) in both isolated mouse cardiomyocytes and failing human heart left-ventricle trabeculae (preclinical and ex vivo human tissue evidence) [29]. Zhang et al. further demonstrated in patients with severe chronic HF that PAGln levels were elevated compared with controls and that gut microbiota composition was substantially altered [30]. The evidence positions PAGln as a candidate for inclusion in future multipathway gut metabolite panels. Whether it is the strongest such candidate requires explicit comparative criteria that have not yet been applied.

Short-chain fatty acids (SCFAs), primarily butyrate, acetate, and propionate, represent a potentially protective counterpart to the potentially harmful TMAO–PAGln axis, though the characterization of SCFAs as potentially cardioprotective remains based primarily on preclinical data. Produced by microbial fermentation of dietary fiber, SCFAs may strengthen the intestinal barrier, attenuate systemic inflammation, and regulate vascular tone and cardiac remodeling via GPR41, GPR43 (also known as FFAR2), and GPR109A (also known as HCAR2) signaling [31]. In murine models, SCFA supplementation reduced blood pressure, attenuated cardiac hypertrophy and cardiorenal fibrosis, and modulated the cardiac immune microenvironment (preclinical evidence) [31]. Clinical studies have consistently found SCFA-producing microbes and SCFA biosynthetic pathways to be depleted in chronic HF [32], though whether this depletion is a cause or consequence of HF progression is not established by observational data alone.

Protein-bound uremic toxins, particularly indoxyl sulfate (IS) and p-cresyl sulfate (PCS), are gut-derived tryptophan and tyrosine metabolites that converge on the cardiorenal axis through mechanisms complementary to TMAO. IS and PCS accumulate in CKD due to high protein binding, which renders them resistant to dialytic clearance, and exert direct cardiac toxicity through ASK1 (apoptosis signal-regulating kinase 1)-MAPK/NF-kB pathways [33] and the AHR (aryl hydrocarbon receptor)–CYP1B1 (cytochrome P450 family 1 subfamily B member 1) axis, disrupting cardiac mitochondrial function and inducing myocardial apoptosis in animal models [34]. Imazu et al. demonstrated in patients with chronic HF that plasma IS levels were independently associated with cardiovascular events over five years of follow-up (human observational evidence) [35]. Zhang et al. identified IS produced by E. coli through the tryptophanase pathway as a key mediator in CKD-related HF and demonstrated that probiotic reduction of E. coli abundance improved cardiac outcomes in both rats and in a small clinical pilot study in CKD patients. This pilot human evidence requires replication in adequately powered randomized trials before it can be considered clinical evidence for probiotic therapy (Table 3) [34].

## 6. The Renal Confounding Problem

The most consistent analytical challenge across the TMAO–HF literature is the attenuation of TMAO prognostic associations following renal function adjustment. This pattern does not appear to be a simple statistical artifact, but reflects genuine biological complexity that resists a single causal interpretation.

**Figure 3:**
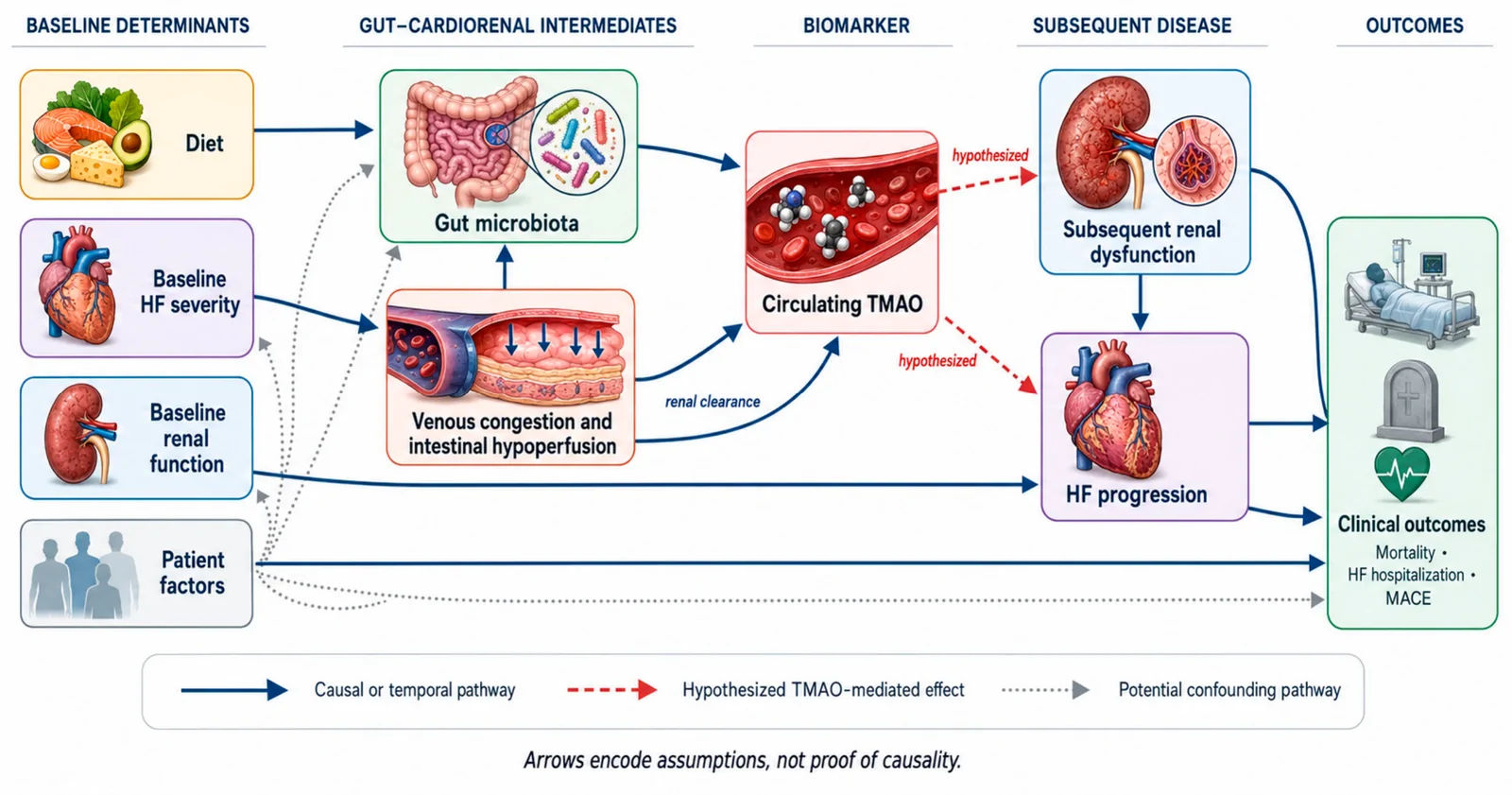
Conceptual directed acyclic graph of TMAO in heart failure.

Renal function may operate in multiple distinct roles in the TMAO–HF relationship: as a confounder, if kidney dysfunction independently increases both TMAO concentrations and HF risk; as a mediator, if TMAO contributes to kidney injury that subsequently worsens HF; as a marker of congestion and overall disease severity; as a time-varying factor influenced by diuretics, hemodynamics, and treatment; or potentially as a collider under specific model structures. Adjustment for eGFR may therefore attenuate the association through a mixture of confounding and possible overadjustment, but the relative contribution of these mechanisms cannot be resolved from observational evidence alone. Figure 3 illustrates these alternative causal relationships as a conceptual directed acyclic graph framework.

In the Leicester AHF cohort, severe multicollinearity between TMAO and renal indices (variance inflation factor 16.2 for eGFR) inflated standard errors and eliminated statistical power to detect independent effects [13]. This is a methodological problem distinct from causal mediation. Tang et al. in CHS/MESA explicitly framed eGFR as either a confounder or a mediator of the TMAO–HF relationship [19], and presented both adjusted models to allow interpretation under either assumption. An important additional limitation of eGFR in this context is that creatinine-based equations may systematically underestimate true glomerular filtration rate in patients with severe cachexia, sarcopenia, or fluid overload, which are common in hospitalized HF populations, potentially introducing residual confounding by renal impairment even in fully adjusted models.

The Li et al. meta-analysis pooled seven prospective studies encompassing 6879 HF patients and reported that higher TMAO was associated with greater MACE risk (HR 1.68) and all-cause mortality (HR 1.67) [36]. The association persisted after eGFR adjustment, but with significant heterogeneity (I2 = 76%), leading the authors to conclude that the TMAO–HF association is only partially mediated by renal dysfunction. This is the only pooled quantitative estimate of the renal mediation question currently available. Its high heterogeneity warrants caution in interpreting the pooled estimate as representative of a consistent effect.

Andrikopoulos et al. applied machine learning and mediation analysis in 1741 Europeans from the MetaCardis study, finding that kidney function was the primary variable predicting circulating TMAO, with gut microbiota composition and diet playing minor, but statistically significant roles [37]. Mediation analysis was consistent, with a bidirectional relationship between TMAO and kidney function, though observational mediation analysis cannot establish causality. Patients receiving SGLT2 inhibitors and GLP-1 receptor agonists had lower circulating TMAO compared with propensity score-matched controls in this observational analysis. Whether this reflects a direct drug effect on gut microbiota, an indirect effect via renoprotection, or residual confounding by indication cannot be determined from a propensity-matched observational analysis. Mendelian randomization studies using genetic instruments for gut microbial TMA production capacity would substantially clarify this causal architecture.

## 7. Population-Specific Variation in TMAO Prognostication

Substantial variation in absolute TMAO levels and their prognostic calibration across populations represents one of the most clinically important challenges in translating gut metabolite biomarkers to global clinical practice. This variation reflects a complex interplay of dietary patterns, gut microbiota composition, renal function, assay platform, fasting status, medication use, and sampling conditions. Aattributing differences primarily to ethnicity or ancestry risks oversimplifying a multifactorial phenomenon.

Japanese patients with HF had approximately twofold higher TMAO concentrations than Caucasian patients in the Yazaki cohort (9.9 vs. 5.9 µmol/L) [15], not fourfold as previously stated. Fish consumption, which provides preformed TMAO directly without requiring microbial conversion, likely contributes to this elevation, but direct dietary measurement was not performed in the outcome cohorts and this attribution is inferential. Geographic variation within the same ancestral group has been demonstrated within BIOSTAT-CHF across 11 European countries [7], likely reflecting regional differences in diet and gut microbiota composition.

The Yazaki tri-ethnic analysis demonstrated that TMAO was associated with death or rehospitalization in Caucasian, but not in South Asian or Japanese patients at equivalent absolute thresholds [15]. Formal interaction tests did not demonstrate statistically significant heterogeneity between groups. The apparent differences in significance may reflect differences in sample size, comorbidity profile, renal function distribution, or dietary patterns rather than genuine ancestry-specific biology. South Asian patients had higher rates of ischemic heart disease and diabetes, suggesting a different dominant pathophysiological substrate. Tang et al. in CHS/MESA reported stronger associations among Black and Hispanic/Latino versus White adults, though again interaction tests were not significant [19]. These observations collectively indicate that universal prognostic thresholds derived from predominantly Caucasian cohorts may not apply across all populations, and population-specific validation studies are needed before any threshold is deployed clinically. Current evidence does not yet establish stable population-specific reference values.

## 8. Therapeutic Implications and Future Directions

The therapeutic landscape of the gut–heart–kidney axis spans several mechanistic levels with very different evidence bases. A staged translational framework—preclinical proof of concept, early human pharmacology, biomarker modulation, safety, and outcome trials—helps contextualize where each approach currently stands.

Dietary modification represents the most immediately accessible intervention. Reduction in red meat, egg, and animal product consumption is associated with lower circulating TMAO in observational and controlled feeding studies, and plant-based dietary patterns are associated with reduced TMA-generating microbial capacity [38]. However, dietary patterns affect multiple cardiometabolic pathways simultaneously, and lower TMAO in the context of a plant-based diet cannot be attributed solely to TMAO reduction. Fish consumption represents a well-recognized complexity: fish elevates circulating TMAO through preformed dietary TMAO, yet is associated with favorable cardiovascular outcomes in epidemiological studies. Differences in dose, food matrix, co-nutrients such as omega-3 fatty acids, renal handling, and residual confounding are plausible explanations for this apparent inconsistency and should be investigated before inferring that preformed and endogenously generated TMAO are biologically distinct molecules.

Pharmacological targeting of the gut–TMAO axis has demonstrated preclinical efficacy across multiple models. 3,3-Dimethyl-1-butanol (DMB), a structural analogue of choline, inhibits gut microbial TMA lyase and reduces circulating TMAO in rodents, attenuating atherosclerosis and reducing renal tubulointerstitial fibrosis in animal models [6]. Organ et al. demonstrated in a murine transverse aortic constriction model that pharmacological inhibition of gut microbial choline TMA lyase with iodomethylcholine improved left ventricular function and attenuated adverse remodeling [24]. Gupta et al. and Zhang et al. further demonstrated preclinical renal protection with iodomethylcholine in murine CKD models [39,40]. Both DMB and iodomethylcholine remain preclinical tools: human pharmacology, safety, selectivity, and microbiome-wide off-target effects have not been characterized in clinical studies. FMO3 inhibition represents another mechanistic node, but carries significant metabolic risks given FMO3’s broader role in sterol and xenobiotic metabolism.

Patients receiving SGLT2 inhibitors and GLP-1 receptor agonists had lower circulating TMAO compared with propensity-matched controls in the MetaCardis observational analysis [37]. This observational finding does not demonstrate that these drugs directly reduce TMAO: lower TMAO may reflect renoprotection, gut barrier effects, weight loss, dietary changes, or residual confounding by indication. Whether any TMAO reduction with these agents mediates part of their established cardiovascular benefit in HF is an important hypothesis addressable through biomarker substudies of existing large HF trials such as EMPEROR-Reduced and DAPA-HF, but it remains untested.

Measurement standardization is a prerequisite for clinical translation. Current studies employ stable-isotope dilution LC–MS/MS, UPLC–MS, and NMR spectroscopy with different internal standards and calibration approaches, generating inter-laboratory variation that substantially limits direct comparability of effect estimates across the studies reviewed. No certified reference materials for TMAO or related gut metabolites and no international harmonization initiative analogous to the IFCC working group for cardiac troponin have been established to the authors’ knowledge. The specific numerical estimates for intra-assay coefficients of variation and inter-laboratory discrepancies cited in earlier versions of this review were not sourced from cited references and have been removed.

## 9. Knowledge Gaps

Key Unresolved Questions: A Research Agenda

1. Is TMAO causal or merely a risk marker? Without human interventional data demonstrating that lowering TMAO improves outcomes, TMAO cannot be classified as a causal mediator rather than a prognostic risk marker. Mendelian randomization studies using genetic instruments for gut microbial TMA production capacity are needed.
2. Is eGFR a confounder, mediator, or both? The Li et al. meta-analysis [36] and the Andrikopoulos MetaCardis data [37] support partial mediation, but formal causal inference testing with Mendelian randomization instruments is needed. Directed acyclic graph analysis applied to existing cohort data would also clarify the causal structure.
3. Which HF phenotype derives greatest prognostic value? TMAO shows more consistent associations in HFrEF. The largest HFpEF-specific dataset (LURIC, n = 395) showed a null result [12], and the two positive HFpEF studies (n = 146 and n = 118) are single-center and hypothesis-generating [14,17]. A prospective multicenter HFpEF cohort with prespecified panel analysis is needed.
4. What is the optimal panel composition? Panels studied to date were assembled empirically and used different metabolites, outcome definitions, and time horizons. A formal optimization study using standardized assays across multiple cohorts, incorporating PAGln and indoxyl sulfate alongside TMAO-pathway metabolites, and reporting C-statistic change and calibration metrics has not been performed.
5. Can lowering TMAO improve cardiovascular outcomes? No human interventional trial has tested this question. This is the single most important gap. Without such evidence, routine clinical TMAO measurement cannot be justified outside research settings.
6. Which thresholds apply across populations? Population-specific validation of prognostic thresholds is needed across ancestral groups, accounting for renal function distribution, assay platform, dietary assessment, and medication use rather than treating ancestry as a surrogate for these factors.
7. Do SGLT2 inhibitors or GLP-1 receptor agonists reduce TMAO, and does this mediate cardiovascular benefit? The MetaCardis observational finding [37] generates this hypothesis. It is testable through biomarker substudies of EMPEROR-Reduced and DAPA-HF.

## 10. Conclusions

The last decade has witnessed a substantive evolution in understanding of TMAO as a cardiovascular biomarker in HF. From its initial characterization as a predictor of adverse outcomes in stable chronic HF, the evidence base now spans acute decompensated HF, incident HF in community cohorts, HFpEF, and ischemic cardiomyopathy across multiple continents and ancestral populations. Four themes dominate the contemporary literature.

First, the prognostic associations of TMAO beyond NT-proBNP, documented in multiple independent cohorts, are of potential clinical relevance because they may capture pathophysiological information from the gut–heart–kidney axis not reflected by conventional biomarkers; however, the magnitude of incremental predictive value, as measured by C-statistic improvement rather than NRI alone, and its clinical utility in guiding treatment decisions remain unestablished. Second, the renal confounding problem remains the central unresolved analytical challenge: adjustment for eGFR may attenuate associations through a mixture of confounding and overadjustment, but the relative contribution cannot be resolved from observational evidence, and creatinine-based eGFR has important limitations in hospitalized HF populations. Third, the temporal dissociation between carnitine-pathway metabolites (potentially useful for acute triage) and TMAO (potentially useful for long-term prognostication) identified in the Israr Leicester dataset [8] has direct potential implications for clinical biomarker strategy, pending external validation. Fourth, neurohormonal GDMT does not modulate TMAO [7], while observational evidence suggests renoprotective agents may be associated with lower TMAO [37]. Whether this represents a mechanistically actionable relationship remains to be tested.

The metabolite panel concept, demonstrated across three independent datasets spanning incident HF in the United States, acute HF in Europe, and acute HF in China, represents an important recent conceptual advance. Selected multimetabolite panels may provide additional prognostic information beyond single-marker TMAO in specific cohorts, and the stepwise mortality gradient with increasing numbers of elevated markers is biologically plausible; however, panels have been assembled empirically, calibration and external validation are lacking, and hazard ratios from differently constructed panels should not be compared directly as measures of predictive performance. The emerging evidence for PAGln, SCFAs, and uremic toxins as parallel gut-derived cardiovascular mediators further broadens the scope of the gut–heart–kidney axis, as summarized in Table 3.

An important limitation of the entire evidence base must be restated: all 14 studies included here are observational (prospective cohorts and cross-sectional), and no human interventional trial has demonstrated that lowering TMAO improves cardiovascular outcomes in HF. Until such evidence exists, TMAO and related metabolites should be understood as prognostic risk markers rather than validated causal targets, and the clinical utility of routine gut metabolite measurement remains to be established. The gut–heart–kidney axis represents a promising, but unvalidated therapeutic framework. What the field now urgently requires is the first adequately powered human interventional trial to test it.

## Figures and Tables

**Table 1:** Characteristics of studies included in this review.

Study (Year)	Country	Study Type	n	HF Type/Setting	TMAO Assay	Primary Endpoint	Follow-Up	Renal Adj.
Tang 2014 [4]	USA	Prospective cohort—established CHF	720	Stable HFrEF, outpatient	SID LC-MS/MS	5-yr all-cause mortality	5 yr	Yes
Trøseid 2015 [5]	Norway	Prospective cohort—established CHF	155 HF, 33 HCs, 100 CAD	Stable HF, NYHA II-IV	SID LC-MS/MS	Transplant-free survival	5.2 yr	Partial
Senthong 2016 [11]	Thailand	Cross-sectional—high CV risk, no HF	134	Stable high-CV-risk, no HF	SID LC-MS/MS	Subclinical myocardial damage (hs-cTnT)	Cross-sectional	Yes
Schuett 2017 [12]	Germany	Prospective cohort—established CHF	823 (428 HFrEF, 395 HFpEF)	HFrEF and HFpEF	LC-MS/MS	All-cause and CV mortality	9.7 yr	Yes (HFrEF only)
Suzuki 2016 [13]	UK	Prospective cohort—acute HF	972	AHF, full EF spectrum	SID UPLC-MS	1-yr mortality and HF rehospitalization	1 yr	No (VIF 16.2)
Suzuki 2019 [7]	11 European countries	Prospective cohort—acute/worsening HF	2234	New/worsening HF, GDMT uptitration	SID UPLC-MS	All-cause mortality	3 yr	Yes
Salzano 2020 [14]	UK	Prospective cohort—established HFpEF	118 HFpEF, 38 HFrEF, 40 HCs	Outpatient HFpEF and HFrEF	SID LC-MS/MS	Composite CV events	18 mo and 5 yr	Yes (threshold)
Yazaki 2020 [15]	UK and Japan	Pooled prospective cohort—acute HF	1087	AHF, tri-ethnic	SID UPLC-MS	1-yr death or HF rehospitalization	1 yr	Caucasian subgroup only
Zhou 2020 [16]	China	Multicenter prospective cohort—ischemic CHF	1208	Post-MI CHF, ischemic HFrEF	SID LC-MS	MACEs	672 days	Yes
Israr 2021 [8]	UK	Prospective cohort—acute HF, panel	806	AHF, 10-metabolite panel	SID UHPLC-MS/MS	30-day and 1-yr death or HF rehospitalization	1 yr	Partial (AcCarn, GBB)
Kinugasa 2021 [17]	Japan	Prospective cohort—established HFpEF	146	Hospitalized HFpEF (EF ≥ 50%)	LC-MS/MS	Cardiac death or HF hospitalization	2.4 yr	Yes (propensity)
Zong 2022 [18]	China	Prospective cohort—established CHF	471 CHF, 485 HCs	Stable CHF vs. controls	LC-MS/MS (TML + TMAO)	CV death or HF hospitalization	2 yr	Yes (adj. for TMAO)
Tang 2024 [19]	USA	Prospective cohort—incident HF, community	11,768	Community-dwelling, no prevalent HF	SID LC-MS/MS	Incident HF	15.9 yr	Yes (mediator model)
Suzuki 2025 [10]	China	Prospective cohort—acute HF, panel	229	AHF, 5-metabolite panel	SID LC-MS/MS	1-yr and 3-yr all-cause mortality	3 yr	Yes (panel adj. eGFR)

Footnote: AHF, acute heart failure; AcCarn, acetyl-L-carnitine; CAD, coronary artery disease; CV, cardiovascular; eGFR, estimated glomerular filtration rate; GBB, gamma-butyrobetaine; HCs, healthy controls; HF, heart failure; HFpEF, HF with preserved ejection fraction; HFrEF, HF with reduced ejection fraction; hs-cTnT, high-sensitivity cardiac troponin T; MACEs, major adverse cardiovascular events; SID, stable-isotope dilution; TML, trimethyllysine; UPLC, ultrahigh-performance liquid chromatography; VIF, variance inflation factor. The Senthong 2016 [11] study is a cross-sectional analysis in high-cardiovascular-risk participants without established HF and is included to characterize the TMAO–myocardial injury relationship in a pre-HF population. It is not counted as a prospective HF outcome cohort.

**Table 2:** Prognostic findings by study in chronological order.

Study	Key Effect Estimate	Renal Adj. Variable Used	Adjustment Result	Key Finding
Tang 2014 [4]	Q4 vs. Q1 HR 3.42 (95% CI 2.24–5.23), 5-yr mortality	eGFR (creatinine)	Retains significance	TMAO + BNP both elevated: 5.7× mortality risk; first CHF cohort
Trøseid 2015 [5]	T3 HR 2.24, transplant-free survival	eGFR (creatinine)	Attenuated to NS after full adjustment	Only TMAO (not choline or betaine) predicts survival; ischemic HF gradient
Senthong 2016 [11]	OR 1.58 for hs-cTnT elevation	eGFR (creatinine)	Survives eGFR adjustment	r = 0.54 with hs-cTnT; cross-sectional; no HF population
Schuett 2017 [12]	HFrEF: HR 2.33 all-cause mortality; HFpEF: NS	eGFR (creatinine)	Survives full adjustment in HFrEF only	Largest HFpEF dataset (n = 395): null result; TMAO + NT-proBNP: HR 10.25 CV death in HFrEF
Suzuki 2016 [13]	HR 1.35 all-cause mortality (1-yr)	eGFR (creatinine); VIF 16.2	Lost after eGFR (severe multicollinearity)	TMAO + NT-proBNP: OR 3.14; adds value to ADHERE, OPTIMIZE-HF, GWTG-HF
Suzuki 2019 [7]	HR 1.40–1.44 mortality	eGFR (creatinine)	Retains after eGFR adjustment	TMAO unchanged by GDMT; high-high serial pattern: HR 2.21; NRI 16.8%
Salzano 2020 [14]	HFpEF HR 3.82 (18-mo); adjusted HR 2.45 (5-yr)	eGFR (creatinine); threshold 5 µmol/L	Survives eGFR adjustment at threshold	Single-center, n = 118 HFpEF; TMAO + BNP identifies highest-risk HFpEF
Yazaki 2020 [15]	Caucasian: *p* < 0.001; S. Asian and Japanese: NS	eGFR (creatinine); Caucasian subgroup only	Adjusted for eGFR in Caucasian subgroup only	Japanese 9.9 vs. Caucasian 5.9 vs. S. Asian 4.5 µmol/L (approximately twofold difference, not four-fold); population-specific validation needed
Zhou 2020 [16]	Q4 HR 1.57 for MACEs after full adjustment	eGFR (creatinine) and hsCRP	Retains after eGFR and hsCRP adjustment	IDI +12.6%; NRI +9.5%; independent of NT-proBNP in post-MI ischemic HF
Israr 2021 [8]	AcCarn AUC 0.744 in-hospital; TMAO HR 1.26 (1-yr)	eGFR (creatinine)	AcCarn and GBB survive; TMAO mostly attenuated	Temporal dissociation: carnitine short-term, TMAO long-term; NRI up to 56.9%
Kinugasa 2021 [17]	Adjusted HR 1.91, cardiac death or HF hospitalization	eGFR (propensity-matched)	Propensity-adjusted for renal function	Single-center, n = 146; malnutrition amplifies TMAO risk in HFpEF
Zong 2022 [18]	TML T3 HR 1.93 composite; HR 3.96 CV death alone	eGFR (creatinine); adjusted including TMAO	Retains including TMAO in model	TML independent of TMAO; TMAVA mechanism distinct from TMAO accumulation
Tang 2024 [19]	Choline HR 1.44; Crotonobetaine HR 1.24; TMAO HR 1.15 (per doubling)	eGFR (creatinine + cystatin C, CKD-EPI)	Choline survives Bonferroni-corrected threshold; TMAO and crotonobetaine attenuated	First race-stratified panel; stronger in Black and Hispanic/Latino vs. White adults
Suzuki 2025 [10]	Panel HR 3.37 (1-yr); HR 2.70 (3-yr); TMAO alone HR 1.78	eGFR (creatinine)	Panel retains after eGFR adjustment	Stepwise gradient 0 to 5 markers; panel associated with higher HR than single TMAO in this cohort; C-statistic not reported

Footnote: AcCarn, acetyl-L-carnitine; GBB, gamma-butyrobetaine; GDMT, guideline-directed medical therapy; HR, hazard ratio; IDI, integrated discrimination improvement; MACEs, major adverse cardiovascular events; NRI, net reclassification improvement; NS, not significant; OR, odds ratio; Q, quartile; T, tertile; VIF, variance inflation factor. All hazard ratio estimates are from the fully adjusted primary model reported in each study. NRI values should be interpreted with caution, as they can appear favorable even when improvements in clinical decision-making are modest. C-statistic changes are provided where reported.

**Table 3:** Comparative summary of gut-derived metabolites implicated in the gut–heart–kidney axis in heart failure.

Metabolite	Dietary Precursor	Microbial Pathway	Renal Dependence	Proposed Cardiac/Renal Mechanism	Evidence Level	HFrEF Evidence	HFpEF Evidence	Acute HF Evidence
TMAO	Choline, L-carnitine, betaine, TML	*cut/gbu* gene clusters -> TMA; hepatic FMO3 oxidation	High—primary renal clearance	Macrophage scavenger receptor upregulation; platelet activation; TGF-beta/Smad3-mediated cardiac fibrosis; renal tubulointerstitial fibrosis	Human observational (multiple cohorts); preclinical mechanistic	Yes (consistent) [4,5,7,12]	Uncertain (LURIC null n = 395; two small positive studies) [12,14,17]	Yes [8,10,13]
Choline	Dietary phosphatidylcholine (egg, meat, dairy)	*cut* pathway -> TMA	Moderate—partially renal	TMAO precursor; independent association with incident HF; choline excess promotes proatherogenic TMAO	Human observational [19]	Not specifically studied	Not specifically studied	Not specifically studied
Crotonobetaine	L-carnitine catabolism	Anaerobic reduction by Enterobacteria	Moderate	TMA precursor; independent association with incident HF after renal adjustment; mechanism incompletely characterized	Human observational [19]	Not specifically studied	Not specifically studied	Not specifically studied
TML (Trimethyllysine)	Protein-bound lysine (meat, dairy)	Carnitine biosynthesis pathway	Moderate	TMAVA derivative inhibits BBOX-dependent carnitine synthesis; accelerates cardiac hypertrophy via FAO impairment	Human observational [18]; animal mechanistic [25]	Yes [18]	Not specifically studied	Not specifically studied
PAGln (Phenylacetylglutamine)	Dietary phenylalanine (meat, legumes)	Phenylacetate-glutamine conjugation by gut bacteria; hepatic conjugation	Low—primarily hepatic/renal conjugation	Alpha-2A, alpha-2B, and beta-2 adrenergic receptor signaling; platelet activation; negative allosteric modulation of beta-2AR; decreased cardiomyocyte sarcomere contraction	Human observational [26,27,28,30]; preclinical mechanistic [29]	Yes [27,28]	Not specifically studied	Not specifically studied
SCFAs (butyrate, propionate, acetate)	Dietary fiber (fermentable carbohydrates)	Microbial fermentation (Firmicutes, Bacteroidetes)	Low—rapidly absorbed and metabolized	GPR41/43/109A signaling; intestinal barrier strengthening; anti-inflammatory; attenuates cardiac hypertrophy and cardiorenal fibrosis in preclinical models	Preclinical mechanistic [31]; human observational (depletion in CHF) [32]	Depleted in CHF (clinical observational) [32]	Not specifically studied	Not specifically studied
Indoxyl sulfate (IS)	Dietary tryptophan (protein-rich foods)	E. coli tryptophanase pathway -> indole; hepatic sulfation	Very high—protein-bound, poor dialytic clearance	ASK1-MAPK/NF-kB pathway; AHR-CYP1B1 axis disrupting mitochondrial function; myocardial apoptosis; renal tubulointerstitial fibrosis	Human observational [35]; animal mechanistic [34]	Yes [35]	Not specifically studied	Not specifically studied
p-Cresyl sulfate (PCS)	Dietary tyrosine and phenylalanine	Microbial fermentation; hepatic sulfation	Very high—protein-bound, poor dialytic clearance	Oxidative stress; endothelial dysfunction; cardiac fibrosis; complements IS on cardiorenal axis	Human observational [33]; preclinical mechanistic	Observational evidence in CKD-HF overlap [33]	Not specifically studied	Not specifically studied

Footnote: AcCarn, acetyl-L-carnitine; AHR, aryl hydrocarbon receptor; ASK1, apoptosis signal-regulating kinase 1; BBOX, gamma-butyrobetaine hydroxylase; beta-2AR, beta-2 adrenergic receptor; CKD, chronic kidney disease; CYP1B1, cytochrome P450 family 1 subfamily B member 1; FAO, fatty acid oxidation; FMO3, flavin-containing monooxygenase 3; GPR, G protein-coupled receptor; HF, heart failure; HFpEF, HF with preserved ejection fraction; HFrEF, HF with reduced ejection fraction; IS, indoxyl sulfate; MAPK, mitogen-activated protein kinase; NF-kB, nuclear factor kappa-light-chain enhancer of activated B cells; PAGln, phenylacetylglutamine; PCS, p-cresyl sulfate; SCFA, short-chain fatty acid; TML, trimethyllysine; TMAO, trimethylamine N-oxide; TMAVA, trimethyl-5-aminovaleric acid.

## Data Availability

No new data were created or analyzed in this study. This manuscript is a narrative state-of-the-art review. All data discussed are available in the cited published studies.
